# Leucovorin Enhances the Anti-cancer Effect of Bortezomib in Colorectal Cancer Cells

**DOI:** 10.1038/s41598-017-00839-9

**Published:** 2017-04-06

**Authors:** Shu Wang, Lei Wang, Zhiyang Zhou, Qipan Deng, Ling Li, Mingzhi Zhang, Linlin Liu, Yong Li

**Affiliations:** 1 Department of Radiotherapy, The Second Hospital of Jilin University, Changchun, Jilin Province 130041 China; 2 Department of Cancer Biology, Lerner Research Institute, Cleveland Clinic, Cleveland, OH 44195 USA; 3 Key Laboratory of Ecology and Environmental Science in Guangdong Higher Education, Guangdong Provincial Key Laboratory for Healthy and Safe Aquaculture, College of Life Science, South China Normal University, Guangzhou, 510631 China; 4 Department of Oncology, The First Affiliated Hospital of Zhengzhou University; Lymphoma Diagnosis and Treatment Center of Henan Province, Zhengzhou, Henan Province 450000 China

## Abstract

Colorectal cancer is a major cancer type worldwide. 5-fluorouracil, often given with leucovorin, is the most commonly used drug in colorectal cancer chemotherapy, yet development of drug resistance to 5-fluorouracil in colorectal cancer cells is the primary cause of chemotherapy failure. Most patients receiving intravenous 5-fluorouracil develop side effects. Leucovorin, due to its vitamin-like profile, has few side-effects. Drug repurposing is the application of approved drugs to treat new indications. In this study, we performed a novel drug-repurposing screening to identify Food and Drug Administration-approved chemotherapeutic compounds possessing synergistic activity with leucovorin against colorectal cancer cells. We found that the combination of bortezomib and leucovorin enhanced caspase activation and increased apoptosis in colorectal cancer cells better than either agent alone. Further, the synergistic induction of apoptosis and inhibition of tumor growth were also observed in mouse colorectal cancer xenografts. These data support leucovorin enhances the anti-cancer effect of bortezomib and present this novel combinatorial treatment against colorectal cancer.

## Introduction

Colorectal cancer (CRC) is the third most common malignant tumor and the fourth most common cause of cancer deaths worldwide^1^. CRC is treated through surgery combined with radiation and/or chemotherapy, depending on the tumor site and disease stage^2, 3^. The standard cytotoxic chemotherapy regimens for CRC patients are FOLFOX containing leucovorin calcium (also known as folinic acid, FOL), 5-fluorouracil (5-FU), and oxaliplatin and FOLFIRI containing FOL, 5-FU, and irinotecan hydrochloride, FOLFIRI)^4–6^. Despite an initial clinical response rate of 40–50% is achieved, a large portion of CRC tumors eventually develop resistance to 5-FU^7, 8^. Therefore, there is an unmet clinical need for novel therapeutic agents or new combination treatments to achieve CRC remission.

Leucovorin itself has no intrinsic cytotoxic activity. Leucovorin is a 5-formyl derivative of tetrahydrofolic acid that is converted to other reduced folic acid derivatives (e.g., tetrahydrofolate) and thus has vitamin activity equivalent to that of folic acid. Leucovorin enhances 5-FU antitumor activity by inhibiting thymidylate synthase in tumor cells^9, 10^. 5-FU incurs common adverse effects, including inflammation of the mouth, loss of appetite, low blood cell counts, hair loss, and inflammation of the skin. On the other hand, due to its vitamin-like properties, leucovorin is generally considered safe and has fewer side effects than 5-FU, oxaliplatin, and other cytotoxic agents used in CRC chemotherapeutic regimens. We hypothesize that beyond 5-FU, leucovorin has synergistic anti-cancer activity against CRC with other chemotherapeutic agents. In this study, we performed a small-scale drug screening to identify Food and Drug Administration (FDA)-approved oncologic drugs that in combination with leucovorin inhibit CRC cell growth and tumorigenesis. We found that the combination of bortezomib and leucovorin is superior to either agent alone in elevating CRC apoptosis and attenuating tumor growth. These data support a new regimen to treat this deadly malignancy.

## Results

### Bortezomib and leucovorin synergistically inhibited the viability of CRC cells

**Figure 1:**
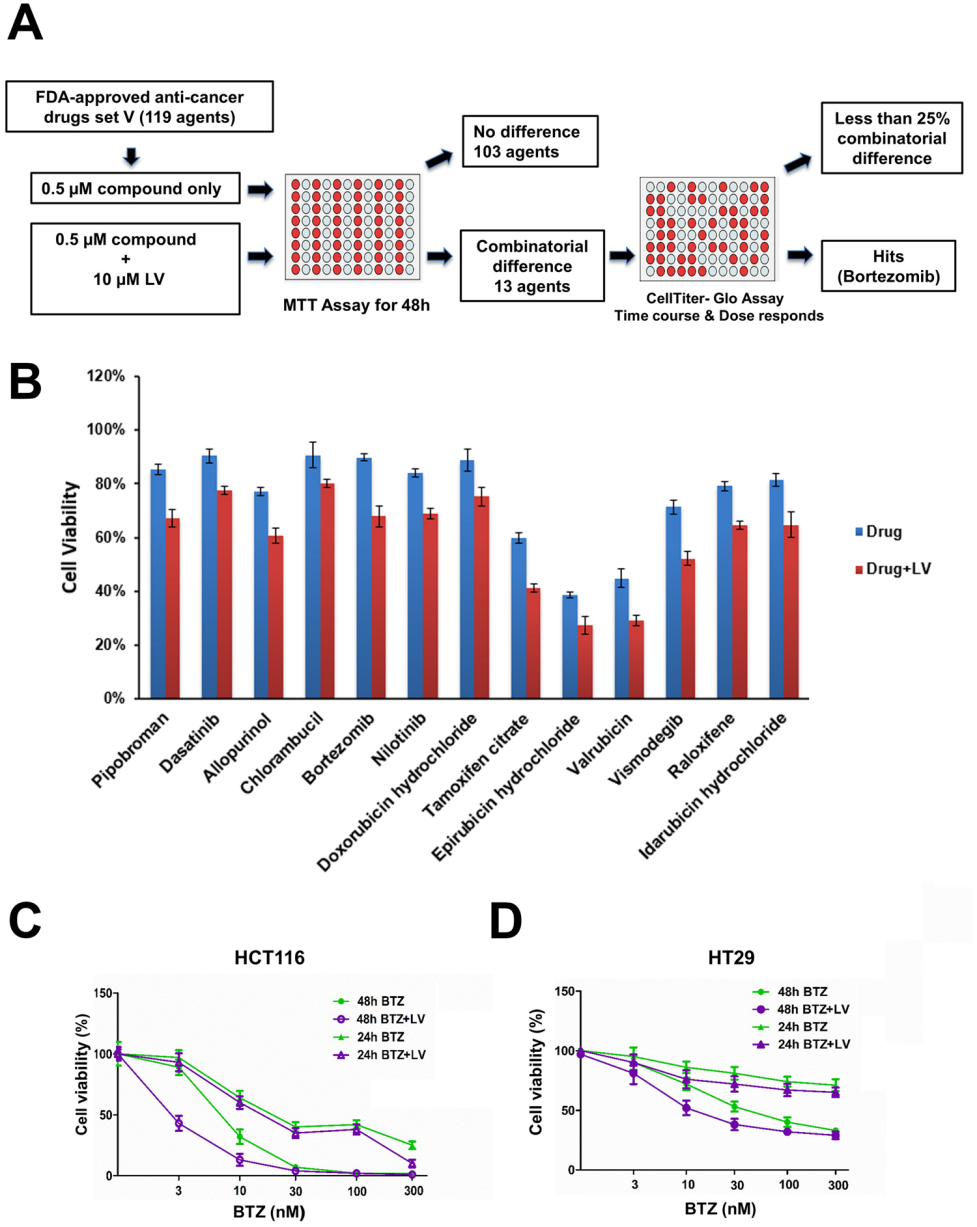
Bortezomib and leucovorin reduce cell viability of HCT116 and HT29 cells. **(A)** Schematic presentation of the screening of the Approved Oncology Drugs Set VI from NCI. **(B)** Cell viability of HCT116 cells treated with a single drug or with the addition of leucovorin. **(C,D)** Percent viability of HCT116 (**B**) and HT29 (**C**) cells upon treatment with the combination of bortezomib (BTZ, 3 nM to 300 nM) and leucovorin (LV, 10 µM) compared to those treated with bortezomib alone for 24 h and 48 h. Cell viability was determined by CellTiter-Glo assays (**C** and **D**).

First, using 3-(4,5-dimethylthiazol-2-Yl)-2,5-diphenyltetrazolium bromide (MTT) assay, we screened FDA-approved anti-cancer drugs set, which contained 119 compounds (https://dtp.cancer.gov/). These compounds were obtained from the Approved Oncology Drugs Set VI at the NCI Developmental Therapeutics Program and were used with a final concentration of 0.5 μM with or without leucovorin (10 μM) to treat human CRC HCT116 cells (Fig. 1A). When combined with leucovorin, 13 compounds had at least a 25% increase in anti-CRC activity, as measured by the MTT assay, compared to these agents alone (Fig. 1B). We then performed time-course and dose-response studies of leucovorin in combination with each of the selected 13 compounds in HCT116 and HT29 CRC cells and assessed viability using the Cell Titer-Glo assay. Only the combination of leucovorin and bortezomib decreased cell viability by more than 25% for both cell lines compared with each compound alone (Fig. 1C,D). The combination of leucovorin and any of the 12 other compounds did not inhibit HT29 cell viability with the 25% improvement. In particular, combinatorial treatment with bortezomib and leucovorin caused synergistic cell death compared with single-drug treatment at 48 h (HCT116, p < 0.01 and HT29, p < 0.05). Combination index (CI) values for each fraction affected were calculated by median drug effect analysis according to the method of Chou and Talalay^11^. In this method, CI < 1.0 indicates synergy; CI = 1.0 indicates an additive effect; and CI > 1.0 indicates antagonism. We found the CI values ranged from 0.50 to 0.85 with leucovorin (10 μM) and three concentrations of bortezomib (3, 10, 30 nM) for both cell lines (Table 1), supporting that the combination of bortezomib and leucovorin exerts a synergistic effect.

**Table 1:** Combination index (CI) values for the combination of bortezomib and leucovorin for HCT116 and HT29 cells.

Bortezomib dose (nM)	Leucovorin dose (µM)	Combination index (CI)*	
		HCT116	HT29
3	10	0.50	0.65
10	10	0.42	0.48
30	10	0.85	0.81

^*^CI > 1, Antagonism; CI < 1, Synergy.

### Bortezomib and leucovorin induced apoptosis in CRC cells

**Figure 2:**
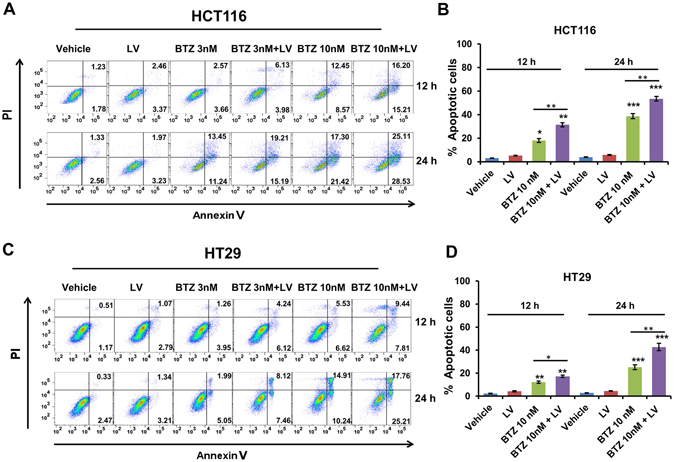
Bortezomib and leucovorin increase apoptosis of CRC cells. (**A,C**) HCT116 and HT29 cells were treated with the combination of bortezomib (3 or 10 nM) and leucovorin (10 µM) or either drug alone for 12 h and 24 h, respectively. The treated cells were stained with FITC-annexin V/propidium iodide, and apoptosis was analyzed with flow cytometry. Experiments were performed three times with one representative experiment shown. Cells that underwent late apoptotic death and early apoptotic death are in the upper and lower right quadrants, respectively. **(B,D)**. Percent apoptotic cells. One-way ANOVA was performed to compare each treatment group with the vehicle control (PBS) or to compare the combination with bortezomib alone. *P ≤ 0.05, **P ≤ 0.01, ***P ≤ 0.001.

We investigated whether the synergistic killing of CRC cells by bortezomib and leucovorin was due to apoptosis. HCT116 and HT29 cells were treated with bortezomib (3 nM or 10 nM), leucovorin (10 μM), or the combination for 12 and 24 h. The treated cells were assayed for apoptosis using annexin V/propidium iodide (PI) staining followed by flow cytometry. Bortezomib plus leucovorin treatment resulted in a higher percentage of apoptotic cells, when compared with either drug alone, in a time- and dose-dependent manner (Fig. 2A,C). Flow cytometry data clearly showed a synergistic induction of apoptosis with the combined treatment. At 24 h, the percentage of apoptotic cells upon treatment with the combination of bortezomib (10 nM) and leucovorin (10 μM) or bortezomib (10 nM) alone increased 15% in HCT116 and 18% in HT29, respectively (Fig. 2B,D). These results suggest that apoptosis induction contributes to the synergistic killing of CRC cells by bortezomib and leucovorin.

### Bortezomib and leucovorin induced G2/M arrest of CRC cells

**Figure 3:**
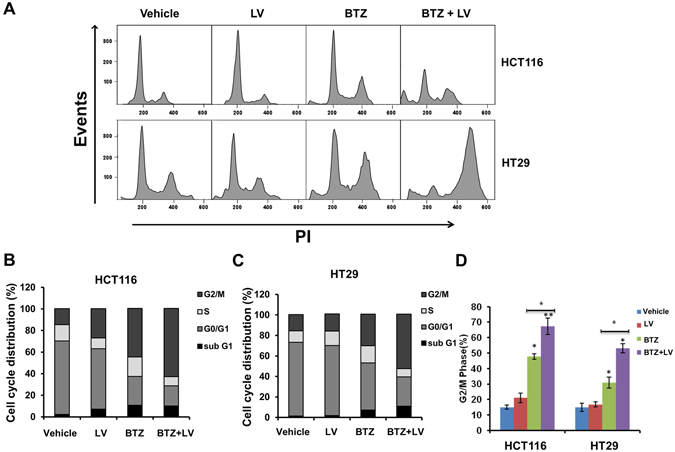
The combination of bortezomib and leucovorin induced G2/M arrest in CRC cells. (**A**) Colon cancer cells were treated with 10 nM bortezomib and/or 10 µM leucovorin or appropriate concentration of vehicle control for 24 h. The cells were harvested, washed with PBS, typsinized, fixed overnight with 70% ethanol at −20 °C, and stained with PI solution before being subjected to flow cytometry. Cell cycle distribution analysis was performed using FlowJo. **(B,C)** Histogram showing the percentage of HCT116 and HT29 cells in the sub-G1, G0-G1, S, and G2/M phases. **(D)** Representative cell cycle histogram demonstrating the percentage of cells in the G2/M phase. *P ≤ 0.05. **P ≤ Each experiment was performed four times, and values are presented as the mean ± SE.

It is reported that bortezomib induces G2/M arrest in human colon cancer cells^12^. To monitor cell cycle, HCT116 and HT29 cells were treated with bortezomib (10 nM) with or without leucovorin (10 μM) for 24 h and subjected to flow cytometry (Fig. 3A). We analyzed the cell cycle distribution for both cell lines using FlowJo (Fig. 3B,C). An increase in the percentage of cells in G2/M phase was observed in both cell lines when treated with bortezomib; adding leucovorin further increased the percentage in G2/M phase (Fig. 3D). These data indicate that leucovorin enhances bortezomib-mediated cell cycle arrest of CRC cells.

### Synergistic induction of apoptosis by bortezomib and leucovorin is mediated through an increase in caspase activation

**Figure 4:**
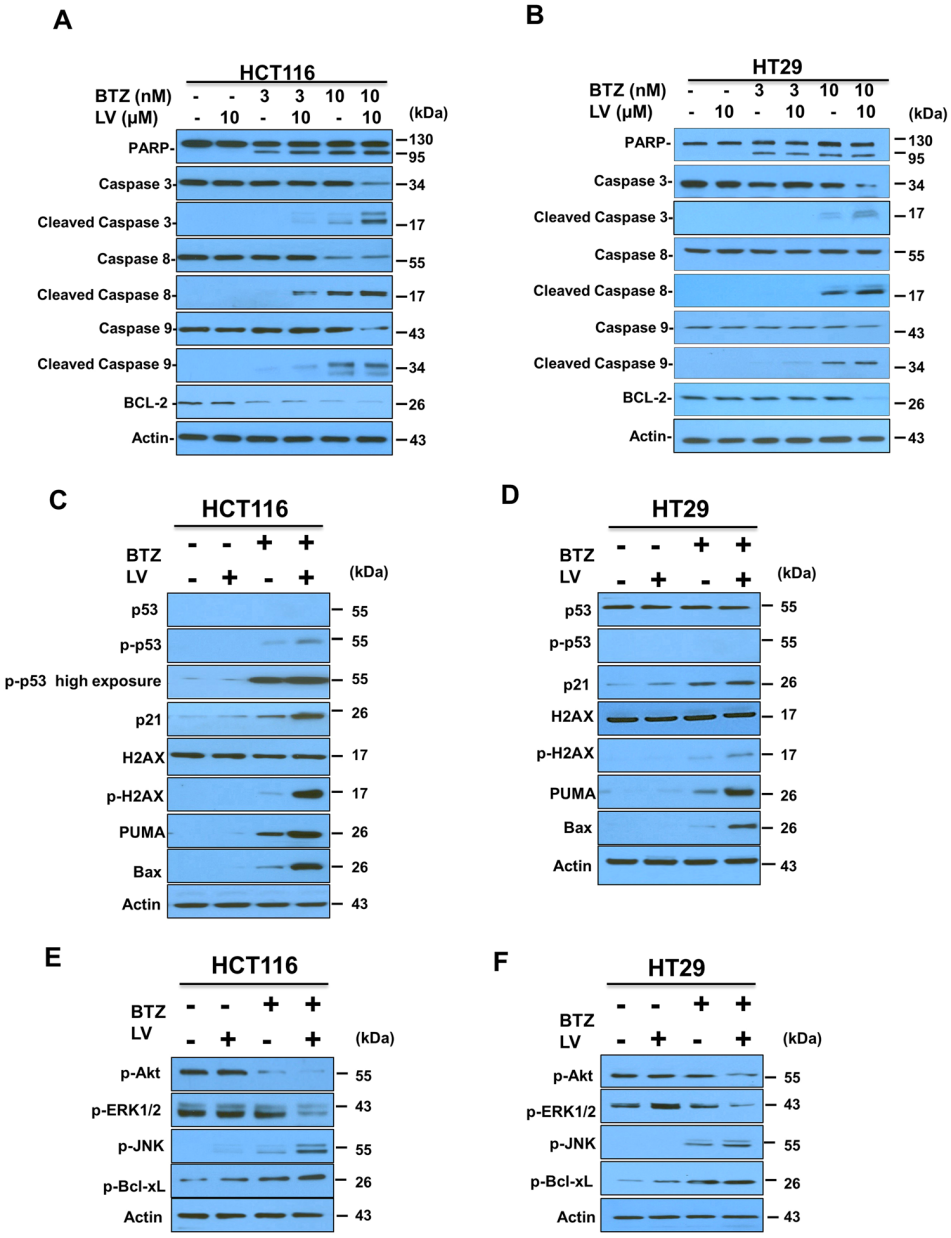
Bortezomib and leucovorin induce activation of caspases and the DNA damage response. HCT116 **(A,C,E)** and HT29 **(B,D,F)** cells were treated with bortezomib (3 or 10 nM) and 10 µM leucovorin or with a single agent for 24 h. Cell lysates were separated by SDS-PAGE and the target protein expression was analyzed by western blotting analysis. Actin was used as a loading control. **(A,B)** PARP and caspases, **(C,D)** p53 and its targets, **(E,F)** kinase signaling.

As the combination treatment enhanced apoptosis, we next examined whether this increased apoptosis is mediated by caspase activation. Using western blotting, we analyzed the proteolytic cleavage of poly (ADP-ribose) polymerase (PARP), caspase-3, -8, -9, and the expression of BCL-2 in both HCT116 and HT29 cells. In HCT116 cells treated with a low dose of bortezomib alone (3 nM), cleavage of PARP, but not the caspases, was detected (Fig. 4A). Adding leucovorin (10 μM) to bortezomib (3 nM) led to cleavage of caspase 8 and 9. With high-dose bortezomib (10 nM) alone, the processing of PARP and caspases -3, -8, and - 9 was readily detectable in HCT116 cells, whereas the combination strongly enhanced caspase-3 processing. BCL-2, a negative regulator of apoptosis, was downregulated in HCT116 cells receiving low- or high-dose bortezomib, but leucovorin did not enhance the downregulation. Similar results were obtained for cleavage of PARP and the caspases in HT29 cells (Fig. 4B). In addition, in HT29 cells BCL-2 downregulation was only observed with high-dose bortezomib (10 nM) plus leucovorin, unlike in HCT116 cells. Overall, these results indicate that leucovorin augments the cleavage of PARP and caspases in bortezomib-induced CRC cell apoptosis.

### Bortezomib and leucovorin induced the activation of DNA damage signaling pathway

It is reported that proteasome inhibitors induce p53-dependent apoptosis in cancer cells^13^. Therefore, we investigated the DNA damage response in HCT116 and HT29 cells by western blotting. In HCT116 cells (which retain a wild-type *TP53* gene)^14^, phosphorylation of p53 at Ser-46 was elevated with bortezomib (10 nM) treatment, while the addition of leucovorin (10 μM) further increased the level of phospho-p53 (Fig. 4C). Next we determined the expression of the p53 transactivational targets, Puma, Bax, and p21, in HCT116 cells and found their expression followed a similar pattern (Fig. 4D).

Phosphorylation of H2AX, a marker of DNA damage and repair, is an early cellular response to the induction of DNA double-strand breaks. This phosphorylation event has emerged as a highly specific and sensitive molecular marker for monitoring DNA damage initiation and resolution^15^. We monitored the expression and phosphorylation of H2AX in HCT116 cells and detected a low level of phospho-H2AX with bortezomib treatment. However, the combination treatment markedly increased H2AX phosphorylation compared with bortezomib alone (Fig. 4C). Beyond CRC cells, bortezomib treatment induces H2AX phosphorylation in gastrointestinal stromal tumor cells and esophageal squamous cell carcinoma cells^16, 17^. However, the exact mechanism underlying such induction is unknown. Increased phosphorylation of Bcl-xL at Ser-62 was observed with combination treatment in both HCT116 and HT29 cells (Fig. 4C,D).

We also determined the expression of p53 and its transactivational targets in HT29 cells. As the p53 gene in HT29 cells is mutated^18^, p53 is neither upregulated nor phosphorylated (Fig. 4D). Yet we found that bortezomib treatment upregulated expression of all three p53 transactivational targets and the upregulation was further enhanced by leucovorin. In addition, bortezomib treatment increased H2AX phosphorylation, which was further augmented by leucovorin in HT29 cells. These results suggest that upregulation of PUMA, Bax, p21, and H2AX phosphorylation may not be dependent on a functional p53 gene, at least in HT29 cells. Taken together, these data imply that cell death induced by bortezomib and leucovorin depends on the activation of pro-apoptotic factors such as PUMA and Bax, anti-apoptotic factors such as phosphorylated Bcl-xL, and increased DNA damage response as judged by H2AX phosphorylation^19^.

### Bortezomib and leucovorin induced the activation of other pathways

Previous studies indicate that mitogen-activated protein kinase (MAPK) pathways play a critical role in the development and progression of cancer^20^. The extracellular signal-regulated kinases (ERKs) and c-Jun N-terminal kinases (JNKs) are two major MAPK classes. ERK is a downstream component of an evolutionarily conserved signaling module, and its activation also promotes an autocrine growth loop critical for tumor growth^21^. JNK activity is tumor suppressive and JNK inhibitors have been considered for cancer therapy due to their ability to promote apoptosis^20^. Western blotting analysis indicated that in HCT116 and HT29 cells, bortezomib alone and to a markedly greater extent combination treatment, inhibited phospho-ERK1/2 expression but activated JNK (Fig. 4E,F). Akt (protein kinase B), is a serine/threonine-specific protein kinase and activated by phosphorylation at Ser-473 that promotes cell growth and confers resistance to apoptosis^22^. We found that Akt phosphorylation was down-regulated in the combinatorial treatment compared with either agent alone (Fig. 4E,F). These results suggest that bortezomib and leucovorin activate JNK signaling, but inhibit the activation of ERK and Akt, suggesting that this combination has multifaceted actions against CRC.

### Bortezomib and leucovorin attenuate HCT116 xenograft tumorigenesis

**Figure 5:**
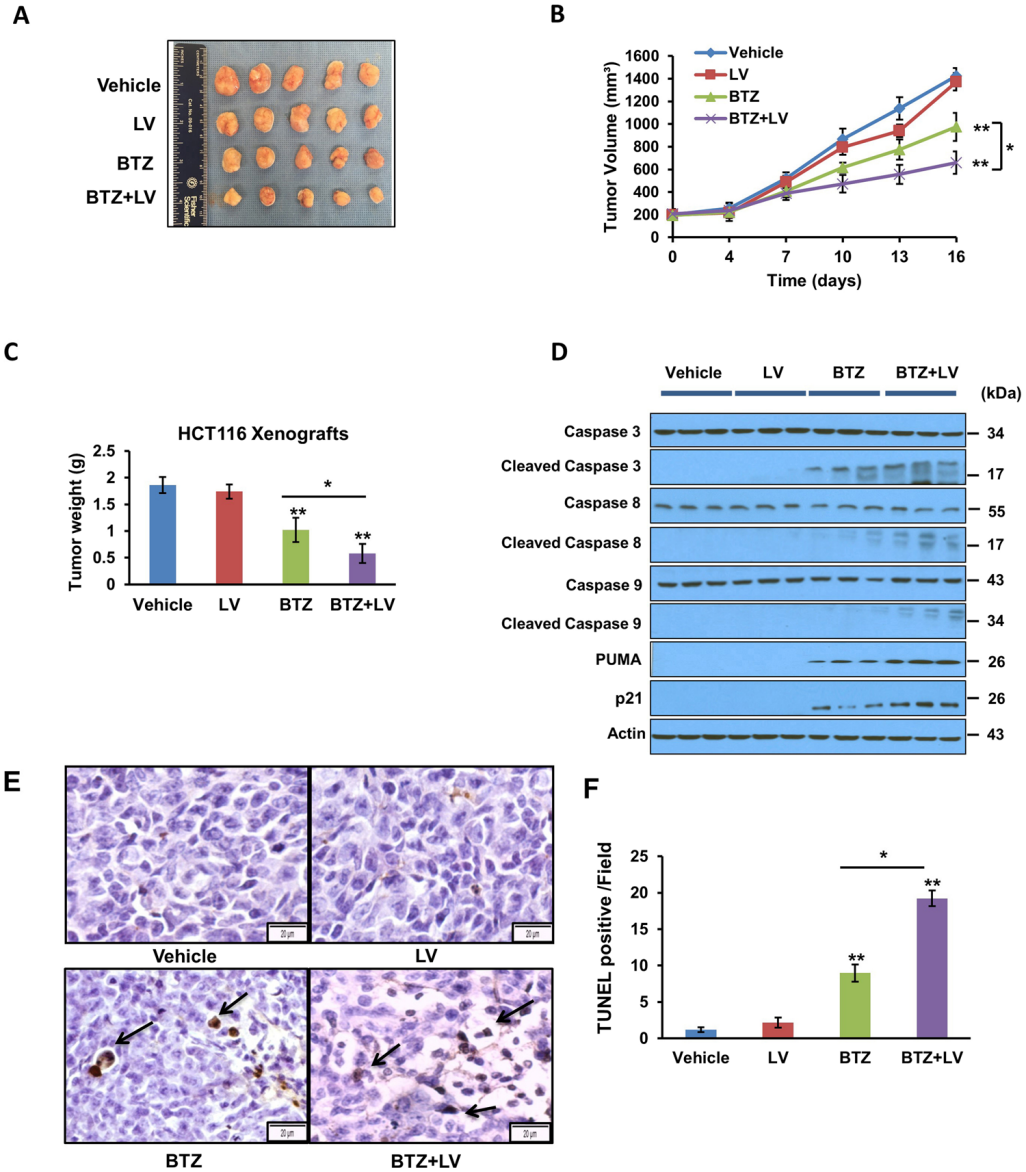
Bortezomib and leucovorin attenuate tumorigenesis of CRC xenografts in mice. HCT116 cells (5 × 10^5^ per mouse) were subcutaneously injected into the right flank of NSG mice. When the tumors reached 200 mm^3^, mice (n = 5 per group) were arbitrarily assigned to one of four groups for treatment with vehicle, 0.5 mg/kg bortezomib, 80 mg/kg leucovorin, or the combination twice a week by intraperitoneal injection. **(A)** Gross tumors at day 16 post treatment. (**B**) Tumor volumes during treatment were measured using a caliper. Data are presented as the mean tumor volume with SEM. (**C**) Tumor weights at day 16. (**D**) Western blot analyses of caspases and their activation (n = 3 representative tumors of 5). (**E**) TUNEL staining to detect apoptosis of tumor cells. A representative image is shown for each group. Arrows points to apoptotic cells. Scale bar = 20 µm. (**F**) Percentage of TUNEL-positive tumor cells per microscopic field. A total of 10 fields was counted for each group. The percentage was calculated as number of cellular nuclei staining positive divided by the total number of nuclei.

Finally, we evaluated the *in vivo* anti-tumor effect of bortezomib and leucovorin on CRC xenografts. HCT116 cells were inoculated subcutaneously on the right flank of immunodeficient NOD scid gamma (NSG) mice. Once tumors were established (tumor volume reached ~200 mm^3^), mice (n = 5 each group) were injected intraperitoneally with the vehicle control (phosphate-buffered saline, PBS), 0.5 mg/kg bortezomib, 80 mg/kg leucovorin, or the combination twice per week. Tumor size and mouse body weight were measured during the treatment. There were no significant differences in animal body weight throughout the study. We found that leucovorin treatment had no effect on tumor growth, but bortezomib treatment suppressed tumor growth significantly (Fig. 5A,B). Moreover, the combination of bortezomib and leucovorin inhibited tumorigenesis to a greater extent than bortezomib alone. Western blotting analyses demonstrated that enhanced cleavage of PARP and caspases (-3, -8, and -9) and activated the expression of PUMA and p21 in tumors resected from NSG mice treated with both agents compared to either agent alone (Fig. 5D). The terminal deoxynucleotidyl transferase dUTP nick end labeling (TUNEL) assay confirmed more apoptotic cell death in xenograft tumor tissues at day 16 after bortezomib and leucovorin combination treatment in comparison to either agent alone or the vehicle control (Fig. 5E,F). These data suggest that bortezomib and leucovorin were able to induce apoptosis and attenuate tumor growth *in vivo*.

## Discussion

Bortezomib (Velcade®, also known as PS-341) is the first proteasome inhibitor approved by the FDA for treating multiple myeloma^23^. Ongoing experimental studies and clinical trials have revealed that, as a single agent or in combination with other conventional anti-cancer drugs^24–26^, bortezomib has inhibitory effects on various types of solid tumors, such as colon and gastric cancers^27–29^, and breast^30–33^, prostate^24, 34, 35^, and lung cancers^36–38^. At present, bortezomib was approved by the FDA for the treatment of patients with multiple myeloma or patients with mantle cell lymphoma. Specific to CRC, we note that several cell culture-based studies demonstrate that bortezomib alone inhibits cell growth and xenograft tumorigenesis^12, 39–45^. Several clinical trials targeting CRC using bortezomib and/or other drugs have been performed or are ongoing (https://clinicaltrials.gov/). One of the questions concerning bortezomib use in CRC therapy is whether this protease inhibiter interferes with other chemotherapeutic agents widely used in today’s standard regimens, specifically FOLFOX and FOLFIRI. Both regimens share two common components: 5-fluorouracil (a cytotoxic compound with significant side-effects)^46^ and leucovorin (a vitamin-like drug with a stellar safety profile)^47^. In this study, we performed a drug-repurposing screening to identify the potent cancer agents already approved by the FDA that reduce CRC cell proliferation in combination with leucovorin. We showed that combining bortezomib and leucovorin could potently induce the killing of CRC cells through a mechanism involving the summative effect of caspase-dependent apoptosis. In combination with leucovorin, bortezomib strongly induced the cleavage of PARP, caspase-3, -8 and -9, and downregulated the anti-apoptosis factor BCL-2. These data indicate that apoptosis is a major downstream event of the two-drug combination that inhibits CRC cell growth and tumorigenesis in mice. This is likely to be accompanied by inhibition of cell cycle progression.

We also found that the combinatorial treatment inhibits the activation of ERK and Akt, two major cancer cell survival pathways, and promotes JNK activation. The phosphorylation of Bcl-xL, a downstream effector of JNK signaling, is enhanced. Previously, it was reported that oxaliplatin, a component of the FOLFOX standard regiment, and bortezomib synergistically activated the JNK-Bcl-xL pathway to induce cell apoptosis in HCT116 cells^39^. Our results demonstrate that leucovorin and bortezomib achieve similar results in both HCT116 cells and xenografts.

Results for the sole trial using bortezomib as a single agent to treat metastatic CRC have been reported^28^. Yet no objective treatment response was observed. One limitation of this trial is that the p53 gene status was not determined, and p53 expression levels were unchanged in the 9 patients in whom it was analyzed^28, 45, 48^. Our study here provides insights into the role of p53 and its transactivational targets in CRC cells treated with bortezomib plus leucovorin. We found that p53 activation as judged by its phosphorylation status is induced in HCT116 cells, which possess a wild-type *p53* gene^14^, but not in HT29 cells, which have a mutant *p53* gene^18^. However, the combination treatment increased the expression of p21, the Bcl-2 family protein PUMA, and the pro-apoptotic Bcl-2 family protein Bax, all of which are p53 transactivational targets. The up-regulation of PUMA and p21 was confirmed in CRC xenografts mouse models. This result suggests that bortezomib and leucovorin together can activate the p53 downstream signaling pathway even when the *p53* gene in the CRC cells is mutated.

We further demonstrated that bortezomib and leucovorin effectively induce apoptosis and suppress the growth of CRC xenografts in immunodeficient mice, indicating that the *in vitro* synergistic action of bortezomib and leucovorin in CRC cells also is achieved *in vivo*. Our data call for a new clinical trial to evaluate bortezomib and leucovorin combinatorial treatment against metastatic CRC. In addition, the mechanism underlying how bortezomib and leucovorin induce the DNA damage response should be investigated.

## Materials and Methods

### Cell lines

Human CRC cell lines HCT116 and HT29 were obtained from the American Type Culture Collection (ATCC, Manassas, VA) and cultured in Dulbecco’s Modified Eagle Medium containing 10% fatal bovine serum (Gibco, NY, USA). Mycoplasma testing was conducted every 3 months to ensure no contamination. All cells were maintained in a humidified incubator at 37 °C and 5% CO_2_. For all studies, CRC cells were grown to ~70% confluence on 10-cm plates and then treated with bortezomib and/or leucovorin calcium for the indicated time periods. All methods related to human cells were carried out in accordance with National Institutes of Health (NIH) guidelines and regulations and Cleveland Clinic Institutional Biosafety Committee polices.

### Reagents

Bortezomib was obtained from Selleck Chemical LLC, Houston, TX; leucovorin calcium was from Sigma-Aldrich, St. Louis, MO. The 119 screened compounds (10 mM in DMSO) are from the Approved Oncology Drugs Set VI at the Developmental Therapeutics Program of the National Cancer Institute. Bortezomib is dissolved in ethanol (200 mg/mL) and leucovorin 40 mg/mL in water before diluted with PBS to treat cells or mice.

### Compounds screening

CRC HCT116 cells were seeded in triplicate in 96-well plates treated with a final concentration of 0.5 μM with or without leucovorin (10 μM) by the MTT assay (ATCC, Manassas, VA). The time-course and dose-response studies of leucovorin in combination with each of the selected 13 compounds in HCT116 and HT29 CRC cells and assessed viability using the Cell Titer-Glo assay (Promega, Madison, Wisconsin).

### Cell viability assay

CRC cells were seeded in triplicate in 96-well plates and treated with bortezomib (0, 3, 10, 30, 100 and 300 nM) and/or leucovorin (10 µM) for 24 or 48 h. Viability was measured using the MTT Cell Proliferation Assay Kit (ATCC, Manassas, VA). Further studies were carried out using the Promega CellTiter-Glo luminescent Cell Viability Assay Kit (Promega, Madison, Wisconsin). Cells were then treated with CellTiter-Glo reagent for 10 minutes at room temperature. Luminescence was determined using a multi-mode plate reader (BioTek).

### Annexin V/propidium iodide assay

CRC cells were incubated with bortezomib (3 or 10 nM) and/or leucovorin (10 μM) for 12 or 24 h. After the incubation, floating and adherent cells were trypsinized, harvested, and washed with serum-free medium, suspended in binding buffer. The cell suspension was stained with FITC-conjugated annexin V and propidium iodide (PI) using the Alexa Fluor 488 annexin V/Dead Cell Apoptosis Kit (Life Technologies, Carlsbad, CA) and then analyzed by flow cytometry. Cell apoptosis and cell cycle distribution analysis were performed using FlowJo.

### Protein extracts and Western blotting analysis

Plates were scraped with 1 × Laemmli lysis buffer (including 2.4 M glycerol, 0.14 M Tris (pH 6.8), 0.21 M SDS, and 0.3 mM bromophenol blue), and the collected cells were boiled for 5 min at 95 °C. Protein concentration was measured with BCA protein assay reagent (Pierce, Rockford, IL, USA). The samples were diluted with 1 × lysis buffer containing 20 mM dithiothreitol, and equal amounts of protein were loaded on 10–15% SDS-polyacrylamide gels(Mini-PROTEAN TGX Precast gels, Bio-Rad, Hercules, California, separated, and transferred onto PVDF membranes. The membrane was blocked with 5% nonfat dry milk in TBS-Tween-20 (0.1%, v/v) for 1 hour and incubated with primary antibody at 4 °C overnight. Horseradish peroxidase–conjugated anti-rabbit or anti-mouse IgG was used as the secondary antibody. Immunoreactive protein was visualized with the Pierce ECL Western blotting substrate (Thermo Scientific, Rockford, IL), according to the provided protocol. Quantitation of X-ray film was carried out by scanning densitometry using area integration. The antibodies were purchased from Cell Signaling Technology (Danvers, MA, USA): caspase-3 (#9662), caspase-8 (#9746), caspase-9 (#9508), cleaved-caspase-3 (#9664), cleaved-caspase-8 (#9496), cleaved-caspase-9 (#9501), PARP (#9542), BCL-2 (#2827), p53 (#2521), p-p53 (Ser46) (#2524), H2AX (#2595), phospho-H2AX (Ser139) (#2577), PUMA (#7467), Bax (#5023), p21 (#2947), phospho-c-Jun NH2-terminal kinase (JNK) (#9251), p-ERK1/2 (Thr202/Tyr204) (#9101), p-Akt (Ser473) (#4062), and β-actin (#3700). Phosphorylated Bcl-xL (S62) antibody was obtained from Millipore (Billerica, MA, USA).

### Animal model

Human colorectal HCT116 xenograft tumors were established by subcutaneously injecting 5 × 10^5^ cells suspended in 1:1 mixture of PBS and Matrigel (Corning, NY, USA) into the right flank of 6-week-old female NSG mice (Jackson, MA, USA). Tumors were measured twice per week using calipers. Tumor volume was calculated as width × length × height × 0.52. Treatment was administered by intraperitoneal injection twice per week when tumor volumes reached ~200 mm^3^. Mice were randomized into four treatment groups (n = 5 per group). For the drug combination, leucovorin was administered 1 h earlier than bortezomib. PBS was used as the vehicle control. Mice were fed ad libitum and maintained in environments with a controlled temperature of ~22 °C and 12 hour light and dark cycles. After 16 days treatment, animal were sacrificed and tumors were assessed. All methods regarding animals were carried out in accordance with NIH guidelines and regulations and experimental protocols were approved by the Cleveland Clinic Institutional Animal Care and Use Committee.

### TUNEL assay

An *in situ* cell apoptosis detection kit (Trevigen, Gaithersburg, MD, USA) was used to detect DNA fragmentation by terminal deoxynucleotidyl transferase dUTP nick end labeling (TUNEL), according to the manufacturer’s instructions. Tissue sections in the vehicle group were stained and served as negative controls. Briefly, sections of paraffin-embedded tissues were deparaffinized, then washed with TBS buffer and permeabilized with proteinase K. DNA strand breaks were then end-labeled with terminal transferase, and the labeled DNA was visualized by fluorescence microscopy.

### Statistical analysis

The significance between the vehicle controls versus treatment groups was determined by one-way analysis of variance (ANOVA) with the appropriate *post hoc* testing using the Tukey’s Test. Statistical analysis was carried out using GraphPad InStat 3 software (GraphPad Software, Inc., San Diego, CA, USA). P ≤ 0.05 was considered statistically significant. Synergism of bortezomib and leucovorin was analyzed with isobologram analysis and combination index (CI) calculation using CompuSyn software (ComboSyn, Inc). CI values below 1 suggest synergy, whereas CI values above 1 indicate antagonism.
